# Prediction of soft tissue sarcoma grading using intratumoral habitats and a peritumoral radiomics nomogram: a multi-center preliminary study

**DOI:** 10.3389/fonc.2024.1433196

**Published:** 2024-12-11

**Authors:** Bo Wang, Hongwei Guo, Meng Zhang, Yonghua Huang, Lisha Duan, Chencui Huang, Jun Xu, Hexiang Wang

**Affiliations:** ^1^ Department of Radiology, The Affiliated Hospital of Qingdao University, Qingdao, Shandong, China; ^2^ Department of Operation Center, Women and Children’s Hospital, Qingdao University, Qingdao, Shandong, China; ^3^ Department of Radiology, The Puyang Oilfield General Hospital, Puyang, Henan, China; ^4^ Department of Radiology, The Third Hospital of Hebei Medical University, Shijiazhuang, Hebei, China; ^5^ Department of Research Collaboration, Research and Development (R&D) Center, Beijing Deepwise and League of Philosophy Doctor (PHD) Technology Co., Ltd, Beijing, China; ^6^ Department of Radiology, Peking University Third Hospital, Beijing, China

**Keywords:** soft tissue sarcoma, habitats, nomogram, radiomics, grade

## Abstract

**Background:**

Accurate identification of pathologic grade before operation is helpful for guiding clinical treatment decisions and improving the prognosis for soft tissue sarcoma (STS).

**Purpose:**

To construct and assess a magnetic resonance imaging (MRI)-based radiomics nomogram incorporating intratumoral habitats (subregions of clusters of voxels containing similar features) and peritumoral features for the preoperative prediction of the pathological grade of STS.

**Methods:**

The MRI data of 145 patients with STS (74 low-grade and 71 high-grade) from 4 hospitals were retrospectively collected, including enhanced T1-weighted and fat-suppressed-T2-weighted sequences. The patients were divided into training cohort (n = 102) and validation cohort (n = 43). K-means clustering was used to divide intratumoral voxels into three habitats according to signal intensity. A number of radiomics features were extracted from tumor-related regions to construct radiomics prediction signatures for seven subgroups. Logistic regression analysis identified peritumoral edema as an independent risk factor. A nomogram was created by merging the best radiomics signature with the peritumoral edema. We evaluated the performance and clinical value of the model using area under the curve (AUC), calibration curves, and decision curve analysis.

**Results:**

A multi-layer perceptron classifier model based on intratumoral habitats and peritumoral features combined gave the best radiomics signature, with an AUC of 0.856 for the validation cohort. The AUC of the nomogram in the validation cohort was 0.868, which was superior to the radiomics signature and the clinical model established by peritumoral edema. The calibration curves and decision curve analyses revealed good calibration and a high clinical application value for this nomogram.

**Conclusion:**

The MRI-based nomogram is accurate and effective for predicting preoperative grading in patients with STS.

## 1 Introduction

Soft tissue sarcomas (STSs) are rare heterogeneous tumors that account for 1% of all tumors (1), and surgical resection is regarded as the primary therapeutic approach for localized STS. Recent studies reported that in addition to surgery, adjuvant radiotherapy and chemotherapy can improve the prognosis for high-grade (grade III) STS. In this respect, the possible adverse consequences of radiation and chemotherapy can be avoided by a preoperative diagnosis of low-grade illness (grades I and II) (2, 3). Accurate preoperative grading is also beneficial for the selection of neoadjuvant chemoradiotherapy (2, 4, 5). Histologic grade is considered to be an important factor affecting the prognosis for STS (4). Although the preoperative histological tumor grading of STS is largely dependent on needle biopsy, because of tumor heterogeneity (6), the initial biopsy-based pathology test may underestimate the actual grade (5, 7). Therefore, there is an urgent need to develop a non-invasive and reliable approach for determining STS grade before surgery, so that patients can receive more effective and targeted treatment.

Magnetic resonance imaging (MRI) is the most commonly used technique for the preoperative diagnosis and evaluation of STS due to its non-invasive nature and excellent soft tissue contrast resolution. Although STS can be diagnosed on MRI by an experienced radiologist, the STS grade is difficult to determine because of tumor heterogeneity (8). Radiomics is a noninvasive method that extracts markers to assist physicians in making judgments through the quantitative mining of features from medical images (9, 10). Considered a digital biopsy, radiomics enables a detailed description of tumor characteristics and spatial heterogeneity in multiple clinical settings (11, 12). In earlier research, biomarkers based on quantitative MRI radiomics features have been regarded as an effective tool to distinguish tumor grades (13, 14). STSs are highly heterogeneous (6), and their growth patterns, tumor heterogeneity, and grade have all been linked in numerous prior investigations (4–7). However, conventional radiomics analysis is usually performed on the whole tumor, thus ignoring regional phenotypic changes within the tumor (15).

Recently, an emerging approach of partitioning tumors into subregions (known as habitats) containing clusters of voxels with similar features has allowed for more effective quantification of intratumor heterogeneity and definition of tumor subregions that are relevant to tumor growth or invasiveness (16–18). A study demonstrated that tumor habitat analysis has high value for predicting tumor grade (19). In addition, recent research demonstrated that the peritumoral microenvironment is valuable for the clinical evaluation of tumor-aggressive biological behavior (20). However, few studies have evaluated the value of both the tumor habitat and the peritumoral environment for accurately predicting the grade and invasive potential of tumors.

Thus, the aim of this study was to develop and validate a non-invasive MRI-based radiomics model combining intratumoral habitats and peritumoral microenvironments for the pretreatment differentiation of high-grade STS from low-grade STS.

## 2 Materials and methods

### 2.1 Study population

This retrospective investigation obtained ethical approval and written informed consent was not required. The inclusion and exclusion criteria are shown in **Supplementary A1** in **Supplementary Data Sheet 1**. Consecutive patients with soft tissue sarcomas treated between January 2007 and July 2022 at the Puyang Oilfield General Hospital, the Third Hospital of Hebei Medical University, the Shandong Provincial Hospital affiliated with Shandong First Medical University, and the Affiliated Hospital of Qingdao University were collected. A total of 145 patients (56 ± 16, 55.2% male) meeting the criteria were enrolled in the study and were divided into two groups. There were 102 patients from the affiliated hospital of Qingdao University in the training cohort and 43 patients from the other three hospitals in the validation cohort. The pathological findings are shown in **Supplementary Table S1**.

### 2.2 MRI protocol

The MRI sequences and scanners are shown in **Supplementary A2** in **Supplementary Data Sheet 1**. The parameters of the MRI sequences are listed in **Supplementary Table S2**.

### 2.3 Clinical information and collection of MRI morphological characteristics

Clinical information including age, sex, and the tumor-node-metastasis (TNM) staging were collected. The FNCLCC (Fédération Nationale des Centres de luttte contre le cancer) system scores for the tumor mitotic index, degree of differentiation, and degree of necrosis were added to give the tumor grade. Histopathological low grade was assigned to FNCLCC grades I and II, whereas histopathological high grade was assigned to grade III.

MRI morphological characteristics were assessed by two radiologists with more than 7 years of experience who were unaware of the pathological findings (**Supplementary A3** in **Supplementary Data Sheet 1**).

### 2.4 Image preprocessing and region-of-interest segmentation

**Figure 1:**
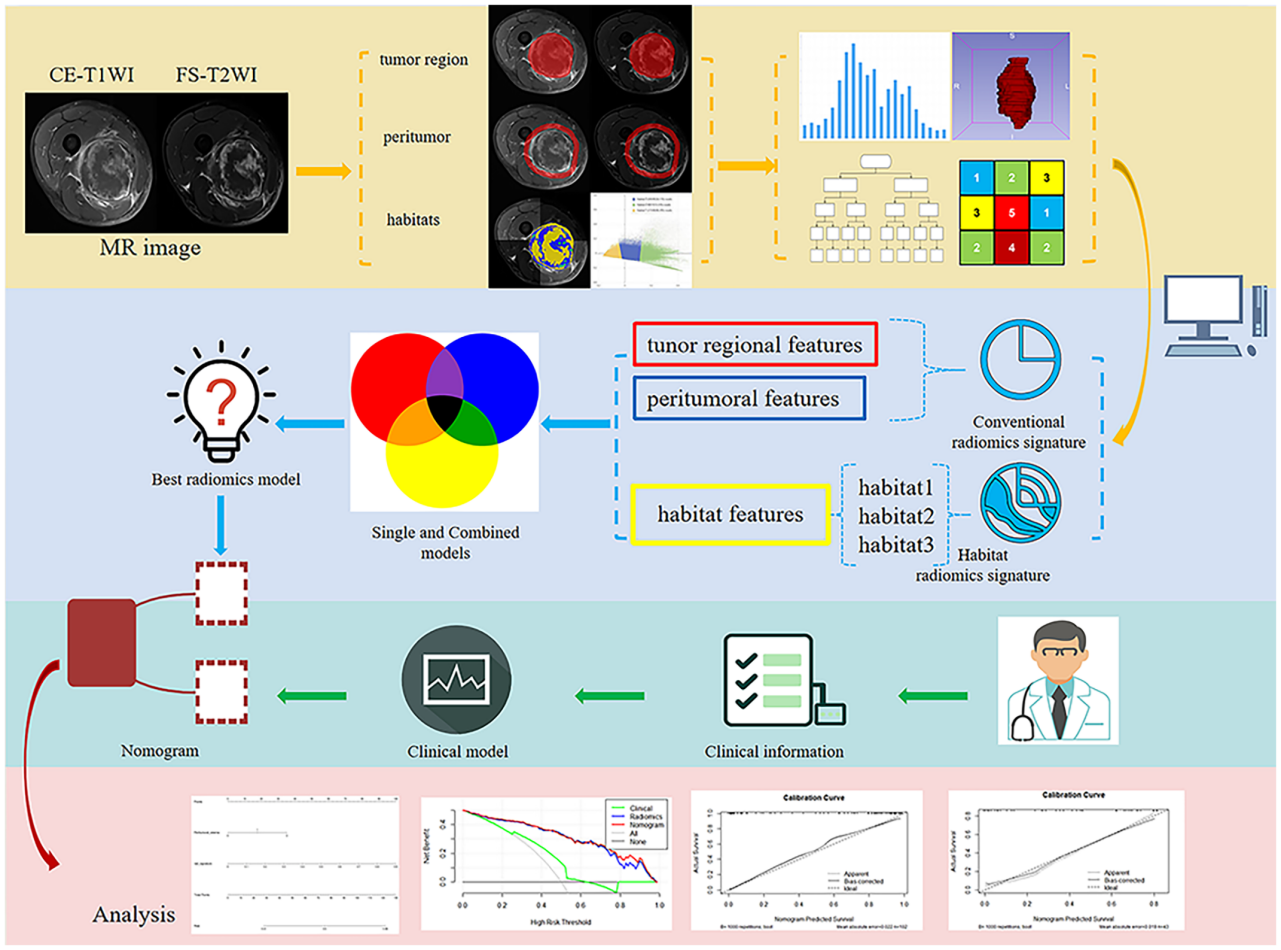
Flowchart illustrating the study design, including data collection, radiomics feature extraction, model training, and validation.

The MRI preprocessing and tumor region-of-interest (ROI) segmentation consisted of four main phases: MRI registration, bias-field correction, segmentation of tumor-associated regions, and spatial resampling. First, using 3D slicer software (v. 5.0.3, www.slicer.org), the CE-T1WI and FS-T2WI of each patient were subjected to 3D rigid transformation registration. Following this registration, bias-field unevenness was corrected using the Python N4-bias-field-correction function. Then, the ROI was accurately drawn, including the tumorous and peritumoral regions, as shown in **Figure 1**. Two primary radiologists manually segmented the tumor ROIs using ITK-SNAP software (v.3.8.0, http://www.itksnap.org). When there were disagreements over an ROI, it was reviewed and corrected by another senior expert radiologist, and the tumor area mask was formed. Then, Radiomics Intelligent Analysis Software (RIAS) was applied to expand the boundary of the tumor lesion mask 10 mm outward for each lesion to generate a peritumoral mask. The “tail sign” can be considered a sign of infiltration or fascial invasion and is an independent factor affecting patient prognosis. It had the same signal intensity as the main mass and had the same enhancement after the injection of gadolinium-based contrast material. Thus, the “tail sign” was segmented into the tumor area mask (21, 22). Large blood vessels, bone tissue, and air areas that had not been invaded in the peritumoral mask were removed manually. Finally, RIAS was used to resample all of the images and masks to an isotropic spatial voxel size of 1 × 1 × 1 mm^3^.

The signal intensity of the CE-T1WI and FS-T2WI was standardized using the normalizing technique for histogram intensity in Python before performing the intratumoral habitats analysis (23). The K-means clustering module in Python was used to cluster the voxels in the normalized CE-T1WI and FS-T2WI images into three clusters representing the functionally-coherent subregions of the STS intratumoral regions, i.e., the intratumoral habitats: a low-enhancement solid subregion with low signal intensity (Habitat 1), a high-signal intensity enhanced viable subregion (Habitat 2), and a low-activity subregion with intermediate signal intensity (Habitat 3).

### 2.5 Extraction of radiomics features

All radiomics feature extraction was performed using the PyRadiomics toolkit in Python. A total of 1906 radiomics features were extracted from the tumoral region masks and peritumoral masks for each sequence, with these including textural features, shape features, first-order features, and wavelet features. A further 93 radiomics features, including textural features and first-order features, were extracted from each of the three intratumoral habitats of each sequence. This resulted in final totals of 558 habitat features, 3812 tumor region features, and 3812 peritumoral features.

### 2.6 Image normalization and standardization method

The combat compensation method (24), which eliminates scanner and protocol influences while maintaining the salient features of texture patterns, was used to standardize the MR images and radiomics features. Then, all features were standardized to Z-scores according to the mean and standard deviation.

### 2.7 Feature selection

Minimum Redundancy Maximum Relevance (mRMR) was used to initially reduce the dimensionality of high-dimensional features, with this resulting in the retaining of 25 highly correlated and low-redundancy features. Then, to further reduce dimensionality and save the features with the greatest predictive ability, we employed the logistic regression method known as the least absolute shrinkage and selection operator (LASSO).

A total of seven subgroups of radiomics signatures were established, with these being the tumoral region signature subgroup, intratumoral habitats signature subgroup, peritumor signature subgroup, signature subgroup combining tumoral region and intratumoral habitats features (TH-combined signature), signature subgroup combining tumoral region and peritumor features (TP-combined signature), signature subgroup combining intratumoral habitats and peritumor features (HP-combined signature), and signature subgroup combining all radiomics features (THP-combined signature). The radiomics features selected for each predictive signature are shown in **Supplementary Table S3**.

### 2.8 Classifiers

Different radiomics signatures to predict STS grade were constructed and evaluated using the following 11 machine learning classifiers: logistic regression (LR), extremely randomized trees (ExtraTrees), support vector machine (SVM), NaiveBayes, K nearest neighbor (KNN), AdaBoost, random forest, eXtreme Gradient Boosting (XGBoost), Multi-Layer perceptron (MLP), GradientBoosting, and Light Gradient Boosting Machine (LightGBM). The classifiers were trained using 10-fold cross-validation applied to the training cohort, and the grade prediction performance of the classifiers was evaluated using area under the receiver operating characteristics (ROC) curve (AUC), accuracy, sensitivity, and specificity. The best machine-learning algorithm was determined to be the one with the highest AUC.

### 2.9 Adding the clinical model and constructing the nomogram

In the construction of the clinical model, the clinical information and MRI morphological characteristics connected to STS grading were first selected using univariate logistic regression. Subsequently, those clinical features with P < 0.05 were entered into a multivariate logistic regression, and peritumoral edema was selected to create clinical models. We then employed the Akaike information criterion-based likelihood ratio test to determine the factors related to STS grading. Finally, we selected the best radiomics signature and combined it with the peritumoral edema to build a nomogram. AUC and accuracy were used to evaluate the performance of the nomogram, radiomics signatures, and clinical model. Calibration curves and decision curve analysis (DCA) were conducted to assess the fit and clinical dependability of the models, respectively.

### 2.10 Statistical analysis

Statistical tests were conducted using SPSS 26.0 (IBM, New York, USA), Python (version 3.9.7, www.python.org), and R software 4.1.2 (https://www.r-project.org/). Continuous variables in the clinical data were analyzed using either independent sample *t*-tests or Mann-Whitney U tests, while categorical variables were analyzed using the chi-square test or Fisher’s exact test. The statistical significance level was set at P < 0.05 for all statistical tests.

## 3 Results

### 3.1 Clinical factors and modeling

The total 145 patients were divided into the training set and the external validation set. The clinical information and MRI characteristics of the patients with STS in the two sets are listed in **Table 1**. There were significant differences in age, depth, heterogeneous SI on FS-T2WI, and location (P < 0.05) between the two sets. Furthermore, no significant differences were observed in the remaining characteristics.

**Table 1:** Patient clinical information and MRI characteristics in the training and validation cohorts.

		Training cohort	Validation cohort	P
No. of patients		102	43	
grade	Low	47	27	0.066
	High	55	16	
Clinical information				
Age *		56 ± 16	47 ± 17	0.002
Sex	Male	52	28	0.118
	Female	50	15	
MRI features				
Depth	Deep	31	25	0.002
	Superficial	71	18	
Number	Single	80	33	0.823
	Many	22	10	
Margin definitions at CE-T1WI	<50%	10	9	0.192
	50%–90%	47	17	
	≥90%	45	17	
Tumor volume containing necrosis signal	Areas without necrosis	30	8	0.031
	1%–50%	56	20	
	>50% of tumor volume	16	15	
Heterogeneous SI at T2WI	<50%	63	12	<0.001
	≥50%	39	31	
Peritumoral enhancement	(+)	50	18	0.430
	(-)	52	25	
Peritumoral edema	No	21	9	0.429
	Limited	72	27	
	Large	9	7	
Location	Limb	77	18	<0.001
	Head and neck	10	4	
	Internal trunk	9	15	
	Trunk wall	6	6	
T-stage	1	21	7	0.749
	2	34	16	
	3	18	10	
	4	29	10	
N-stage	0	85	35	0.778
	1	17	8	
M-stage	0	83	35	0.997
	1	19	8	

No. of Patients: Total number of patients in each cohort; Grade: Tumor grade (Low *vs*. High); MRI Features: Various MRI characteristics such as depth, number, margin definitions, tumor volume containing necrosis signal, heterogeneous SI at T2WI, peritumoral enhancement, peritumoral edema, location, T-stage, N-stage, and M-stage; * Data are presented as mean ± standard deviation.

The results of the univariate and multivariate LR analysis showed that peritumoral edema was an independent predictor of STS grade. **Table 2** shows the univariate and multivariate logistic regression results with P < 0.05. A clinical model was established with the inclusion of peritumoral edema. The AUC values for the clinical model applied to training and validation sets were 0.665 (95% CI: 0.582–0.749) and 0.613 (95% CI: 0.472–0.755), respectively.

**Table 2:** Positive results of clinical information and MRI characteristics in univariate and multivariate logistic regression.

Variable	Univariate			Multivariate		
	OR	95% CI	p value	OR	95% CI	p value
Age	1.010	0.985-1.035	0.439			
Sex	1.381	0.632-3.017	0.418			
Depth	1.660	0.709-3.882	0.243			
Number	0.818	0.318-2.104	0.677			
Margin definitions at CE-T1WI	1.445	0.784-2.665	0.239			
Tumor volume containing necrosis signal	2.054	1.093-3.861	0.025	1.531	0.754-3.106	0.238
Heterogeneous SI at T2WI	2.812	1.211-6.529	0.016	1.815	0.721-4.567	0.205
Peritumoral enhancement	1.599	0.730-3.503	0.240			
Peritumoral edema	4.755	1.868-12.104	<0.001	3.970	1.497-10.524	0.006
Location	1.254	0.832-1.889	0.280			
T stage	1.452	1.009-2.090	0.045			
N stage	1.708	0.579-5.040	0.332			
M stage	1.595	0.571-4.452	0.373			

Variable: Clinical and MRI characteristics; Univariate: Odds ratio (OR), 95% confidence interval (CI), and p-value for each variable in univariate analysis; Multivariate: Same metrics for multivariate analysis, showing the adjusted effects of each variable.

### 3.2 Radiomics feature selection and radiomics signature performance

**Figure 2:**
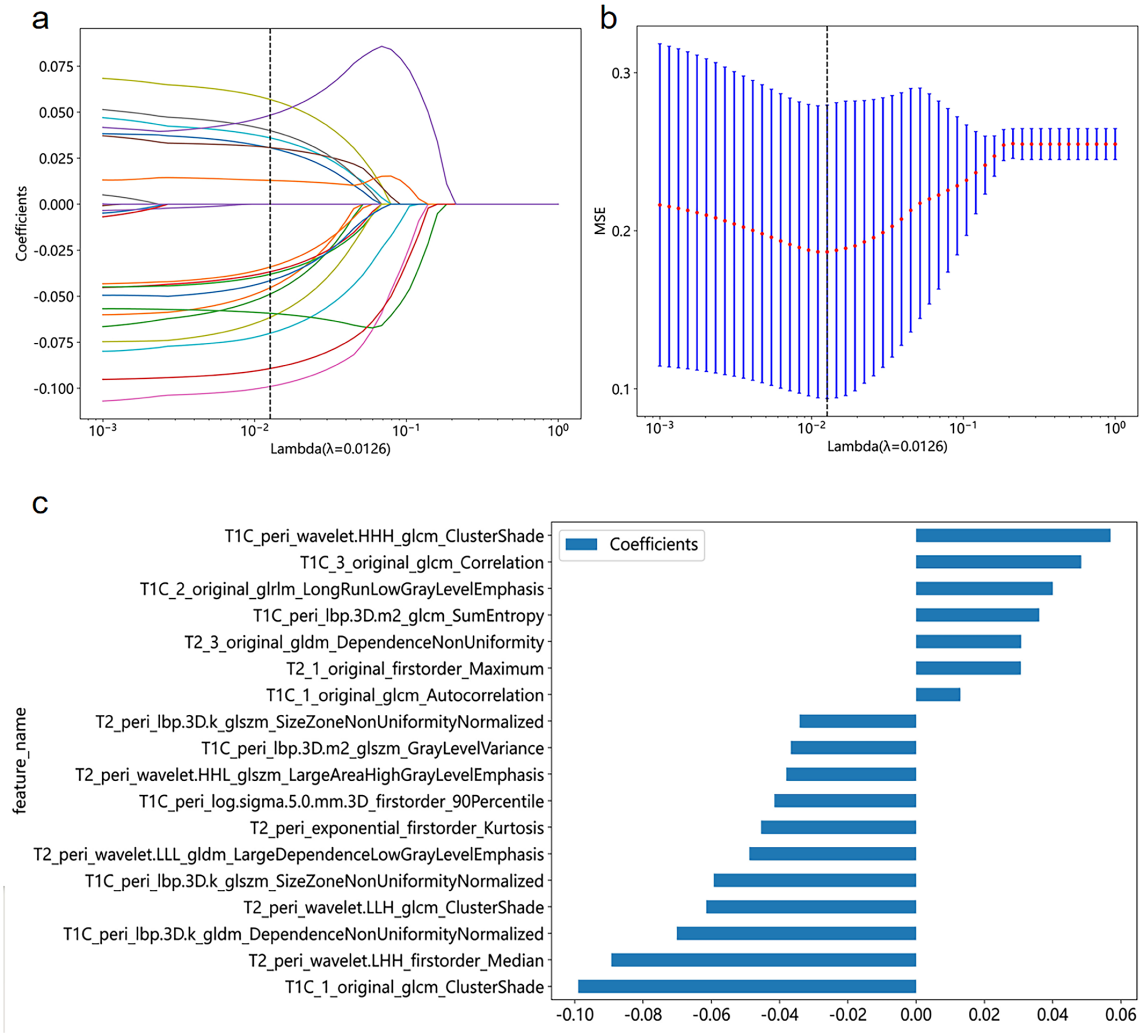
Coefficient profile plot **(A)**, cross-validation plot **(B)**, and histogram of feature weights **(C)** for the best radiomics features selected in the study.

We initially identified 25 features using mRMR and then further screened these features using LASSO. We established seven radiomics signature subgroups and used 11 machine learning methods to construct separate signatures for each subgroup, resulting in a total of 77 radiomics signatures. The AUC and accuracy of these are shown in **Table 3**. For the validation set, the best signature and the optimal performance within each subgroup were as follows: the tumoral region signature built using the ExtraTrees classifier had a highest AUC value of 0.613; the intratumoral habitats signature built using the KNN classifier had a highest AUC value of 0.626; the peritumor signature built using the MLP classifier had a highest AUC value of 0.828; the TH-combined signature built using the AdaBoost classifier had a highest AUC value of 0.589; the TP-combined signature built using the LR classifier had a highest AUC value of 0.738; the HP-combined signature built using the MLP classifier had a highest AUC value of 0.856; and the THP-combined signature built using the MLP classifier had a highest AUC value of 0.657. In summary, we found that the HP-combined signature built by combining mRMR and LASSO with the MLP classifier had the highest predictive performance (for the training and validation sets the accuracy values were 0.873 and 0.814, respectively, and the AUC values were 0.923 and 0.856), and was therefore the model used in the subsequent studies. Eighteen features were selected by LASSO to establish the HP-combined signature (the best radiomics signature), as shown in **Figure 2**, with this including 12 peritumoral features and six habitat features.

**Table 3:** The predictive performance of different radiomics machine learning signatures in the training and validation cohorts.

		Training cohort			Validation cohort		
		ACC	AUC	95% CI	ACC	AUC	95% CI
Tumor region	LR	0.892	0.932	0.884 - 0.988	0.512	0.581	0.405 - 0.757
	ExtraTrees	1.000	1.000	1.000 - 1.000	0.605	0.613	0.446 - 0.781
	SVM	0.922	0.944	0.894 - 0.994	0.651	0.583	0.408 - 0.759
	NaiveBayes	0.882	0.920	0.865 - 0.974	0.651	0.602	0.427 - 0.777
	KNN	0.814	0.903	0.848 - 0.957	0.535	0.591	0.425 - 0.758
	AdaBoost	0.951	0.989	0.976 - 1.000	0.581	0.547	0.369 - 0.726
	RandomForest	0.990	1.000	0.999 - 1.000	0.558	0.574	0.399 - 0.749
	XGBoost	1.000	1.000	1.000 - 1.000	0.674	0.472	0.285 - 0.660
	MLP	0.882	0.916	0.862 - 0.971	0.581	0.574	0.397 - 0.752
	GradientBoosting	0.961	0.996	0.990 - 1.000	0.558	0.497	0.312 - 0.682
	LightGBM	0.892	0.940	0.898 - 0.983	0.465	0.436	0.257 - 0.615
Habitats	LR	0.814	0.831	0.7511 - 0.9116	0.581	0.495	0.317 - 0.674
	ExtraTrees	1.000	1.000	1.000 - 1.000	0.488	0.568	0.398 - 0.739
	SVM	0.804	0.832	0.746 - 0.919	0.605	0.569	0.398 - 0.741
	NaiveBayes	0.775	0.800	0.713 - 0.886	0.581	0.454	0.277 - 0.631
	KNN	0.765	0.843	0.769 - 0.916	0.535	0.626	0.464 - 0.789
	AdaBoost	0.863	0.931	0.886 - 0.976	0.535	0.522	0.344 - 0.700
	RandomForest	0.980	0.997	0.991 - 1.000	0.535	0.602	0.432 - 0.772
	XGBoost	1.000	1.000	1.000 - 1.000	0.581	0.604	0.434 - 0.774
	MLP	0.784	0.831	0.751 - 0.910	0.628	0.609	0.440 - 0.778
	GradientBoosting	0.941	0.977	0.955 - 0.100	0.581	0.521	0.345 - 0.697
	LightGBM	0.804	0.869	0.798 - 0.941	0.488	0.538	0.359 - 0.718
Peritumor	LR	0.863	0.892	0.829 - 0.956	0.764	0.729	0.566 - 0.892
	ExtraTrees	1.000	1.000	1.000 - 1.000	0.628	0.712	0.560 - 0.864
	SVM	0.882	0.960	0.928 - 0.991	0.791	0.799	0.665 - 0.933
	NaiveBayes	0.784	0.821	0.735 - 0.907	0.674	0.799	0.647 - 0.950
	KNN	0.755	0.854	0.787 - 0.921	0.512	0.675	0.519 - 0.831
	AdaBoost	0.902	0.974	0.950 - 0.999	0.605	0.587	0.403 - 0.770
	RandomForest	0.990	1.000	0.999 - 1.000	0.674	0.670	0.505 - 0.835
	XGBoost	1.000	1.000	1.000 - 1.000	0.628	0.641	0.475 - 0.808
	MLP	0.843	0.914	0.860 - 0.968	0.744	0.828	0.719 - 0.947
	GradientBoosting	0.882	0.991	0.980 - 1.000	0.628	0.778	0.639 - 0.917
	LightGBM	0.804	0.926	0.880 - 0.973	0.605	0.609	0.434 - 0.784
Tumor region+ habitats	LR	0.892	0.942	0.900 - 0.984	0.465	0.530	0.352 - 0.709
	ExtraTrees	1.000	1.000	1.000 - 1.000	0.465	0.470	0.293 - 0.647
	SVM	0.912	0.962	0.919 - 1.000	0.558	0.586	0.410 - 0.761
	NaiveBayes	0.794	0.908	0.853 - 0.963	0.605	0.523	0.346 - 0.701
	KNN	0.863	0.919	0.870 - 0.969	0.512	0.573	0.399 - 0.747
	AdaBoost	0.951	0.987	0.972 - 1.000	0.605	0.589	0.411 - 0.768
	RandomForest	0.990	1.000	1.000 - 1.000	0.512	0.495	0.319 - 0.672
	XGBoost	1.000	1.000	1.000 - 1.000	0.488	0.461	0.284 - 0.637
	MLP	0.873	0.940	0.898 - 0.983	0.535	0.562	0.388 - 0.737
	GradientBoosting	0.922	0.994	0.986 - 1.000	0.419	0.517	0.337 - 0.698
Tumor region+ peritumor	LightGBM	0.853	0.954	0.918 - 0.989	0.535	0.579	0.405 - 0.753
	LR	0.882	0.961	0.930 - 0.993	0.698	0.738	0.585 - 0.892
	ExtraTrees	1.000	1.000	1.000 - 1.000	0.674	0.698	0.525 - 0.871
	SVM	0.922	0.969	0.934 - 1.000	0.651	0.716	0.556 - 0.877
	NaiveBayes	0.843	0.905	0.848 - 0.962	0.628	0.699	0.532 - 0.866
	KNN	0.814	0.910	0.859 - 0.962	0.651	0.666	0.506 - 0.825
	AdaBoost	0.941	0.996	0.990 - 1.000	0.628	0.613	0.437 - 0.790
	RandomForest	0.980	1.000	1.000 - 1.000	0.605	0.615	0.445 - 0.784
	XGBoost	1.000	1.000	1.000 - 1.000	0.605	0.566	0.383 - 0.749
	MLP	0.863	0.940	0.897 - 0.982	0.628	0.688	0.525 - 0.851
	GradientBoosting	0.971	0.998	0.994 - 1.000	0.558	0.601	0.426 - 0.776
	LightGBM	0.873	0.963	0.934 - 0.993	0.558	0.546	0.367 - 0.726
Habitats+ peritumor	LR	0.863	0.912	0.857 - 0.967	0.674	0.639	0.466 - 0.812
	ExtraTrees	1.000	1.000	1.000 - 1.000	0.605	0.716	0.561 - 0.872
	SVM	0.912	0.956	0.915 - 0.996	0.674	0.771	0.630 - 0.912
	NaiveBayes	0.833	0.867	0.794 - 0.939	0.651	0.653	0.483 - 0.822
	KNN	0.833	0.912	0.862 - 0.962	0.581	0.670	0.516 - 0.824
	AdaBoost	0.912	0.976	0.954 - 0.998	0.605	0.682	0.522 - 0.842
	RandomForest	0.990	1.000	0.999 - 1.000	0.767	0.765	0.615 - 0.915
	XGBoost	1.000	1.000	1.000 - 1.000	0.698	0.704	0.541 - 0.866
	MLP	0.873	0.923	0.872 - 0.974	0.814	0.856	0.739 - 0.974
	GradientBoosting	0.971	0.988	0.971 - 1.000	0.674	0.619	0.448 - 0.790
	LightGBM	0.882	0.939	0.894 - 0.984	0.628	0.590	0.417 - 0.764
Tumor region+ habitats+ peritumor	LR	0.882	0.950	0.912 - 0.989	0.535	0.611	0.443 - 0.780
	ExtraTrees	1.000	1.000	1.000 - 1.000	0.535	0.598	0.426 - 0.771
	SVM	0.892	0.976	0.954 - 0.998	0.581	0.627	0.458 - 0.796
	NaiveBayes	0.794	0.908	0.853 - 0.963	0.535	0.551	0.372 - 0.730
	KNN	0.833	0.918	0.869 - 0.967	0.581	0.588	0.422 - 0.754
	AdaBoost	0961	0.989	0.975 - 1.000	0.628	0.609	0.435 - 0.783
	RandomForest	0.990	1.000	1.000 - 1.000	0.465	0.506	0.334 - 0.678
	XGBoost	1.000	1.000	1.000 - 1.000	0.581	0.546	0.371 - 0.722
	MLP	0.863	0.937	0.892 - 0.982	0.674	0.657	0.490 - 0.824
	GradientBoosting	0.951	0.994	0.985 - 1.000	0.581	0.596	0.420 - 0.772
	LightGBM	0.902	0.965	0.935 - 0.996	0.535	0.603	0.433 - 0.773

Accuracy (ACC), area under the curve (AUC), and 95% CI for each machine learning model in the training cohort; Validation Cohort: Same metrics for the validation cohort; Models: Various machine learning algorithms (e.g., LR, logistic regression; ExtraTrees, extremely randomized trees; SVM, support vector machine; KNN, K nearest neighbor; XGBoost, eXtreme Gradient Boosting; MLP, Multi-Layer perceptron; LightGBM, Light Gradient Boosting Machine).

### 3.3 Establishment of a radiomics nomogram

**Figure 3:**
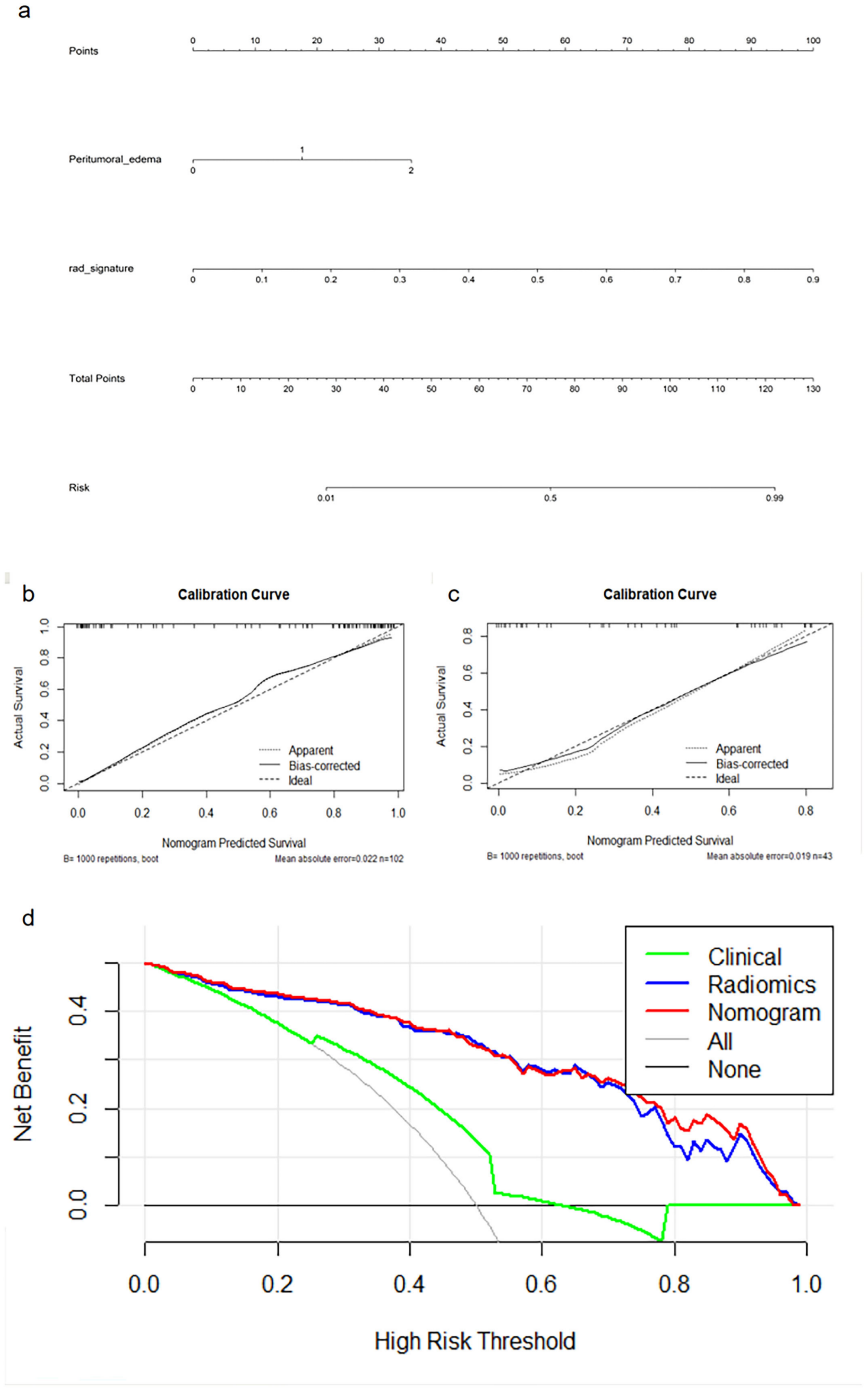
Nomogram **(A)**, calibration curves **(B, C)** of the nomogram in the training and external validation cohorts, and decision curve analysis **(D)** for the nomogram.

A nomogram (**Figure 3A**) combining the best radiomics signature with the clinical independent predictors was subsequently constructed. **Table 4** shows the predictive performance of the radiomics signature, clinical model, and nomogram. The nomogram demonstrated superior performance than the top-performing machine learning signature and clinical model. **Figures 3B–D** illustrates the calibration curves and DCA of the nomogram. The calibration curves showed good calibration in the training set (**Figure 3B**) and confirmed favorable calibration in the validation set (**Figure 3C**), indicating that the nomogram discriminated well. The DCA (**Figure 3D**) demonstrated that the nomogram provided greater clinical application value than the radiomics signature and the clinical model. Consequently, the nomogram should achieve optimal performance in terms of clinical application.

**Table 4:** Predictive performance of radiomics signature, clinical model, and nomogram in the training and validation cohorts.

	Training cohort							Validation cohort						
	ACC	AUC	95% CI	Sensitivity	Specificity	PPV	NPV	ACC	AUC	95% CI	Sensitivity	Specificity	PPV	NPV
Habitats + peri (MLP)	0.873	0.923	0.872 - 0.974	0.964	0.766	0.828	0.947	0.814	0.856	0.739 - 0.974	0.875	0.778	0.700	0.913
Clinical model	0.667	0.665	0.582 - 0.749	0.927	0.362	0.630	0.810	0.535	0.613	0.472 - 0.755	0.938	0.296	0.441	0.889
Nomogram	0.892	0.937	0.892 - 0.981	0.964	0.809	0.855	0.950	0.814	0.868	0.758 - 0.978	0.938	0.741	0.682	0.952

Training Cohort: Accuracy (ACC), AUC, 95% CI, sensitivity, specificity, positive predictive value (PPV), and negative predictive value (NPV) for each model in the training cohort; Validation Cohort: Same metrics for the validation cohort; Models: Radiomics signature (Habitats + peri with MLP), clinical model, and nomogram.

## 4 Discussion

Knowledge of the STS histological grade of a tumor is essential for formulating a therapeutic strategy and is a key factor affecting the prognosis (4, 25). The results of our study showed that a signature based on the radiomics features of the intratumoral habitats and peritumoral microenvironments extracted from preoperative MRI could predict the grade of STS with high accuracy. By analyzing the radiomics features derived from tumorous regions, intratumoral habitats, and peritumoral areas, we established a total of 77 single or combined radiomics signatures from seven different subgroups. Among these, the HP-combined signature established by the MLP classifier combined with mRMR and LASSO feature screening methods yielded the best predictive performance. Finally, the nomogram showed better prediction performance than the radiomics signature and the clinical model based on peritumoral edema, and could thus provide valuable information to clinicians and patients and help guide clinical decision-making. The nomogram calibration curve demonstrated that both cohorts could benefit from the approach, and the DCA confirmed that the nomogram achieved the best clinically applicable performance.

Several qualitative MRI characteristics have previously been reported as potential imaging biomarkers for STS grading. Zhang et al. (26) found peritumoral hyperintensity to be an independent risk factor for predicting histopathological grade. Crombé et al. (4) confirmed that high-grade STS was linked to the MRI morphological characteristics of heterogeneity, necrosis, and peritumoral enhancement. In addition, some studies have shown that in addition to the general MRI features related to the prognosis of STS, some specific STS subtypes are independent prognostic factors for specific STS subtypes, such as the “Tail sign” of undifferentiated pleomorphic sarcoma, the “Water−like” appearance of myxofibrosarcoma, the “Triple sign” and absence of calcifications in synovial sarcoma, signal heterogeneity in myxoid liposarcoma (22). In our study, we included peritumoral edema to establish the clinical model. The prediction performance of the clinical model was poor, with an AUC value of only 0.665 for the training set and 0.613 for the external validation set. This result indicates that the MRI characteristics and the low-dimensional clinical data only capture a small portion of the relevant information present in the imaging data and omit a great deal of the lesion heterogeneity detail.

One key aspect of our study is that we analyzed intratumoral habitats. Zhou et al. proposed the concept of extracting quantitative features from distinct tumor sub-regions (27), and later studies showed that sub-regional radiomics analysis methods may better quantify the tumorous subregion related to tumorous growth or aggressiveness than conventional radiomics, and obtain prediction models with higher accuracy (17, 19). These studies suggest that intratumoral habitats may provide valuable clues for tumor prediction and prognosis. In our study, to obtain a model for predicting STS grade with high accuracy, we considered integrating high-throughput radiomics feature analysis with voxel-based habitat segmentation to predict STS grade. The results showed that among the optimal signatures for each subgroup, the predictive performance of the intratumoral habitats radiomics signature was better than that of the conventional tumor radiomics signature, and the predictive performance of the HP-combined signature was better than that of the TP-combined signature. These results demonstrate that the intratumoral clustered segmented habitats contain important information for STS grading and are capable of predicting STS grade with higher accuracy than conventional radiomics approaches based on the whole tumor.

Another key aspect of our study is that we analyzed the peritumoral area. Clinical evidence demonstrates that the heterogeneity of STS extends beyond the tumor itself, encompassing the surrounding area (28). Consequently, the surrounding environment of the tumor can provide valuable data for assessing the aggressive biological behavior of the tumor. Combinations of intratumoral and peritumoral radiomics features were successfully used in recent research to identify the histological categories of renal cell carcinoma (29) and to differentiate between benign and malignant pulmonary nodules (30, 31). Previous research also revealed that the peritumoral microenvironment provides supplementary information for predicting STS histopathological grade (26). Among the optimal signatures of each subgroup in our study, the peritumor signature outperformed any other single radiomics signature, and the TP-combined signature and HP-combined signature performed better than the single tumoral region signature and intratumoral habitats signature, respectively. These results demonstrate that peritumoral radiomics features provide added predictive value.

In our study, the best-performing HP-combined signature contained six habitat features and 12 peritumoral features, with “T1C 1 original glcm ClusterShade” contributing the most to the performance. This feature was extracted in Habitat 1, which is a low-enhancing solid subregion, and we deduce that this region may have a greater association with tumor grade than the other regions. In terms of feature types, Gray Level Co-occurrence Matrix (GLCM) texture features (classified as higher-order features) have demonstrated significant clinical impacts in both radiology and nuclear medicine (32, 33). Although the blood supply to STS tumors is reflected in the CE-T1WI signal strength, it is difficult to distinguish minor signal intensity changes within tissue on conventional MRI. Specifically, “T1C 1 original glcm ClusterShade” quantifies the skewness and uniformity of gray-scale variability that is imperceptible to visual inspection. Previous studies showed GLCM features to possess strong predictive capabilities for tumor grade and found that they played an indispensable role in the construction of radiomic signatures (34, 35).

Regarding the selection of features in this study, mRMR is a novel method for feature screening that screens radiomics features by employing more rational coefficients and reducing redundancy (36). In comparison, LASSO is a feature screening technique that prevents overfitting during signature construction (37). In this study, 11 machine learning methods (LR, ExtraTrees, SVM, NaiveBayes, KNN, AdaBoost, RandomForest, XGBoost, MLP, GradientBoosting, and LightGBM) were selected for investigation, and in combination with the seven subgroups they resulted in a total of 77 radiomics signatures. MLP is a supervised learning method that can learn nonlinear models in real-time, achieving high accuracy and good generalization ability (38–40). The present results of this study showed that out of all the tested machine learning signatures, the HP-combined signature built by the MLP classifier combined with the popular mRMR and LASSO feature screening techniques demonstrated the highest predictive ability.

There are some limitations to this study. First, it should be noted that this study was conducted retrospectively, which means that although we had strict inclusion and exclusion criteria, there may still have been selection bias. Second, because of the relatively small sample size, the histological subtypes were not distributed evenly, which may have caused some statistical bias. Additionally, our data were collected from four different institutions utilizing similar but not identical scanners and processes. Therefore, to enhance the stability of features, as well as to account for any variations among them, the combat compensation method and a resampling methodology were employed. Finally, manual segmentation was applied to the work, which may lead to deviations. A recent study (41) demonstrated that automatic segmentation achieved favorable performance. This study is only a preliminary analysis, and further research in a larger prospective clinical series is needed to determine associations between patient tumor grade and spatial habitat analysis.

In summary, we developed a nomogram that can accurately and noninvasively predict STS grade before surgery so that patients can obtain more effective and targeted treatment. By enhancing surveillance and improving adjuvant clinical trial design, our predictive model may help close the gap between radiology and precision healthcare.

## Data Availability

The datasets presented in this study can be found in online repositories. The names of the repository/repositories and accession number(s) can be found in the article/**Supplementary Material**.
