# Supplementary material for: Prediction of soft tissue sarcoma grading using intratumoral habitats and a peritumoral radiomics nomogram: a multi-center preliminary study
Source: Front Oncol. 2024 Dec 11;14:1433196. doi: 10.3389/fonc.2024.1433196 (PMC11668965; doi:10.3389/fonc.2024.1433196)
Supplement: Supplementary file 1 [file DataSheet1.docx]

**Supplementary information**

**Supplement A1: The inclusion and exclusion criteria.**

The following inclusion criteria were set: (1) histopathological confirmation of the lesion as STS; (2) MRI examination including contrast-enhanced fat-suppressed T1-weighted imaging (CE-T1WI) and fat-suppressed T2-weighted imaging (FS-T2WI) was conducted 2 weeks before operation; (3) complete medical data were available. The following exclusion criteria were applied: (1) unavailability of complete clinical or imaging information; (2) poor image quality (signal-to-noise ratio ≤ 1.0); (3) preoperative chemotherapy, radiotherapy, immunotherapy, or other treatment; and (4) other unrelated malignancies.

**Supplement A2: The MRI protocol**

The MRI sequences of all patients included the CE-T1WI and FS-T2WI sequences. The MRI scanners used included: Achieva 1.5 T (Philips Healthcare, Amsterdam, Netherlands), Magnetom Skyra 3.0 T (Siemens, Munich, Germany), Prisma (Siemens), and HDx 1.5 T/3.0 T (GE Healthcare, Chicago, IL, USA).

**Supplement A3: The MRI morphological characteristics**

MRI morphological characteristics included: (1) depth (a lesion with a maximum depth of ≥8 cm was considered deep); (2) number (single or many); (3) margin definition on CE-T1WI (<50%, 50%–90%, or ≥90% of the tumor circumference being well-defined); (4) tumor volume containing signal representing necrosis (none, 1%–50%, or ≥50%); (5) heterogeneous signal intensity on FS-T2WI (<50% or ≥50%); (6) peritumoral enhancement (negative, positive); (7) peritumoral edema (none, limited, or large); and (8) location (limb, head and neck, internal trunk wall, or trunk wall).

Table S1. Histopathological confirmation

|  | Training cohort | Validation cohort |
| --- | --- | --- |
|  | (n=102) | (n=43) |
| Myxoid liposarcoma | 17 | 6 |
| Undifferentiated pleomorphic sarcoma | 17 | 1 |
| Solitary fibrous tumor, malignant | 11 | 4 |
| Myxofibrosarcoma | 9 | 3 |
| Dedifferentiated liposarcoma | 7 | 1 |
| Leiomyosarcoma | 7 | 3 |
| Synovial sarcoma | 4 | 5 |
| Pleomorphic liposarcoma | 3 | 2 |
| Malignant peripheral nerve sheath tumor | 1 | 2 |
| Rhabdomyosarcoma | 0 | 5 |
| Extraskeletal osteosarcoma | 4 | 0 |
| Angiosarcoma | 2 | 1 |
| Alveolar soft part sarcoma | 2 | 2 |
| Epithelioid sarcoma | 1 | 0 |
| Spindle cell sarcoma, undifferentiated | 1 | 4 |
| Undifferentiated sarcoma | 8 | 2 |
| Others | 8 | 2 |

Table S2. MRI acquisition parameters.

|  | TE | TR | Slice Spacing | Slice Thickness | Field of View | Matrix |
| --- | --- | --- | --- | --- | --- | --- |
|  | (ms) | (ms) | (mm) | (mm) |  |  |
| CE-T1WI | 10-15 | 500-600 | 1 | 3-5 | 200-400 | 320×320 |
| FS-T2WI | 70-120 | 2400-4500 | 1 | 3-5 | 200-400 | 320×320 |

Note: TE, time of echo; TR, time of repetition; CE-T1WI, contrast enhanced fat-suppressed T1 weighted imaging; FS-T2WI, fat-suppressed T2 weighted imaging.

Table S3. Input features in radiomics signatures.

| Signature | Input features |  |
| --- | --- | --- |
| Tumor region | T1C_reg_logarithm_firstorder_Kurtosis |  |
|  | T2_reg_wavelet.LLL_firstorder_Skewness |  |
|  | T2_reg_square_firstorder_Minimum |  |
|  | T1C_reg_wavelet.HHH_firstorder_Median |  |
|  | T2_reg_squareroot_glcm_ClusterShade |  |
|  | T1C_reg_exponential_gldm_DependenceVariance |  |
|  | T2_reg_wavelet.LHL_firstorder_Mean |  |
|  | T1C_reg_squareroot_glcm_InverseVariance |  |
|  | T2_reg_lbp.3D.m2_glcm_InverseVariance |  |
|  | T1C_reg_log.sigma.4.0.mm.3D_firstorder_Skewness |  |
|  | T2_reg_exponential_firstorder_Median |  |
|  | T2_reg_wavelet.LLH_firstorder_Skewness |  |
|  | T1C_reg_lbp.3D.m2_firstorder_Skewness |  |
|  | T2_reg_squareroot_glszm_LargeAreaHighGrayLevelEmphasis |  |
|  | T1C_reg_wavelet.HLH_glrlm_LongRunHighGrayLevelEmphasis |  |
|  | T2_reg_log.sigma.5.0.mm.3D_firstorder_Skewness |  |
|  | T2_reg_exponential_gldm_DependenceNonUniformityNormalized |  |
|  | T2_reg_lbp.3D.k_glszm_GrayLevelVariance |  |
|  | T2_reg_exponential_glszm_LowGrayLevelZoneEmphasis |  |
| Habitats | T1C_3_original_glcm_Correlation |  |
|  | T1C_1_original_glcm_Autocorrelation |  |
|  | T1C_2_original_glrlm_LongRunLowGrayLevelEmphasis |  |
|  | T2_3_original_gldm_DependenceNonUniformity |  |
|  | T2_1_original_firstorder_Median |  |
|  | T2_1_original_ngtdm_Strength |  |
|  | T1C_1_original_gldm_LargeDependenceHighGrayLevelEmphasis |  |
|  | T2_2_original_glszm_LargeAreaHighGrayLevelEmphasis |  |
|  | T1C_1_original_glcm_ClusterShade |  |
| peritumor | | T1C_peri_lbp.3D.k_glszm_ZoneEntropy |
|  | | T1C_peri_wavelet.HLH_firstorder_Median |
|  | | T1C_peri_lbp.3D.m2_glcm_SumEntropy |
|  | | T2_peri_original_glszm_SmallAreaEmphasis |
|  | | T1C_peri_lbp.3D.m2_firstorder_Range |
|  | | T1C_peri_original_shape_Flatness |
|  | | T1C_peri_lbp.3D.m2_glcm_Correlation |
|  | | T2_peri_lbp.3D.k_glszm_ZoneEntropy |
|  | | T1C_peri_lbp.3D.m2_glszm_HighGrayLevelZoneEmphasis |
|  | | T1C_peri_wavelet.LHL_glcm_Imc2 |
|  | | T1C_peri_lbp.3D.k_glszm_SizeZoneNonUniformityNormalized |
|  | | T1C_peri_lbp.3D.m2_glszm_GrayLevelNonUniformityNormalized |
|  | | T2_peri_log.sigma.1.0.mm.3D_firstorder_Skewness |
|  | | T2_peri_wavelet.LLL_gldm_LargeDependenceLowGrayLevelEmphasis |
|  | | T2_peri_wavelet.LHH_firstorder_Mean |
|  | | T1C_peri_log.sigma.5.0.mm.3D_firstorder_90Percentile |
|  | | T2_peri_wavelet.LHH_firstorder_Median |
|  | | T2_peri_lbp.3D.k_glszm_HighGrayLevelZoneEmphasis |
|  | | T1C_peri_lbp.3D.k_gldm_DependenceNonUniformityNormalized |
|  | | T2_peri_wavelet.HHL_glszm_LargeAreaHighGrayLevelEmphasis |
|  | | T2_peri_wavelet.LLH_glcm_ClusterShade |
|  | | T2_peri_exponential_firstorder_Kurtosis |
| Tumor region  +habitats | T2_reg_wavelet.LLL_firstorder_Skewness |  |
|  | T1C_3_original_glcm_Correlation |  |
|  | T2_reg_square_firstorder_Minimum |  |
|  | T1C_reg_logarithm_firstorder_Kurtosis |  |
|  | T1C_reg_wavelet.HHH_firstorder_Median |  |
|  | T2_reg_wavelet.LHL_firstorder_Mean |  |
|  | T2_reg_squareroot_glcm_ClusterShade |  |
|  | T1C_3_original_firstorder_TotalEnergy |  |
|  | T1C_2_original_glrlm_LongRunLowGrayLevelEmphasis |  |
|  | T2_reg_lbp.3D.m2_glcm_InverseVariance |  |
|  | T1C_1_original_glrlm_LongRunHighGrayLevelEmphasis |  |
|  | T2_1_original_firstorder_Maximum |  |
|  | T2_reg_wavelet.LLH_firstorder_Skewness |  |
|  | T2_reg_log.sigma.5.0.mm.3D_firstorder_Skewness |  |
|  | T1C_reg_lbp.3D.m2_firstorder_Skewness |  |
|  | T2_reg_exponential_gldm_DependenceNonUniformityNormalized |  |
|  | T2_reg_squareroot_glszm_LargeAreaHighGrayLevelEmphasis |  |
|  | T1C_1_original_glcm_ClusterShade |  |
|  | T1C_reg_log.sigma.4.0.mm.3D_firstorder_Skewness |  |
|  | T1C_reg_wavelet.HLH_gldm_LargeDependenceHighGrayLevelEmphasis |  |
|  | T2_reg_exponential_glszm_LowGrayLevelZoneEmphasis |  |
| Tumor region  +peritumor | T1C_reg_logarithm_firstorder_Kurtosis |  |
|  | T2_reg_square_firstorder_Minimum |  |
|  | T2_reg_wavelet.LHL_firstorder_Mean |  |
|  | T1C_reg_exponential_gldm_DependenceVariance |  |
|  | T2_reg_wavelet.LLL_firstorder_Skewness |  |
|  | T2_reg_lbp.3D.m2_glcm_InverseVariance |  |
|  | T2_reg_squareroot_glcm_ClusterShade |  |
|  | T1C_peri_lbp.3D.m2_firstorder_Maximum |  |
|  | T1C_peri_lbp.3D.k_glszm_ZoneEntropy |  |
|  | T1C_reg_log.sigma.4.0.mm.3D_firstorder_Skewness |  |
|  | T1C_reg_wavelet.HLH_glrlm_LongRunHighGrayLevelEmphasis |  |
|  | T1C_reg_lbp.3D.m2_firstorder_Skewness |  |
|  | T2_reg_squareroot_glszm_LargeAreaHighGrayLevelEmphasis |  |
|  | T2_reg_log.sigma.5.0.mm.3D_firstorder_Skewness |  |
|  | T2_reg_lbp.3D.k_glszm_GrayLevelVariance |  |
|  | T1C_peri_bp.3D.k_glszm_SizeZoneNonUniformityNormalized |  |
|  | T2_peri_lbp.3D.k_glszm_HighGrayLevelZoneEmphasis |  |
|  | T2_peri_wavelet.LLH_glcm_ClusterShade |  |
|  | T1C_peri_lbp.3D.k_gldm_DependenceNonUniformityNormalized |  |
|  | T2_reg_exponential_glszm_LowGrayLevelZoneEmphasis |  |
|  | T2_peri_wavelet.LHH_firstorder_Median |  |
| Habitats  + peritumor | T1C_peri_wavelet.HHH_glcm_ClusterShade |  |
|  | T1C_3_original_glcm_Correlation |  |
|  | T1C_2_original_glrlm_LongRunLowGrayLevelEmphasis |  |
|  | T1C_peri_lbp.3D.m2_glcm_SumEntropy |  |
|  | T2_3_original_gldm_DependenceNonUniformity |  |
|  | T2_1_original_firstorder_Maximum |  |
|  | T1C_1_original_glcm_Autocorrelation |  |
|  | T2_peri_lbp.3D.k_glszm_SizeZoneNonUniformityNormalized |  |
|  | T1C_peri_lbp.3D.m2_glszm_GrayLevelVariance |  |
|  | T2_periwavelet.HHL_glszm_LargeAreaHighGrayLevelEmphasis |  |
|  | T1C_peri_log.sigma.5.0.mm.3D_firstorder_90Percentile |  |
|  | T2_peri_exponential_firstorder_Kurtosis |  |
|  | T2_peri_wavelet.LLL_gldm_LargeDependenceLowGrayLevelEmphasis |  |
|  | T1C_peri_lbp.3D.k_glszm_SizeZoneNonUniformityNormalized |  |
|  | T2_peri_wavelet.LLH_glcm_ClusterShade |  |
|  | T1C_peri_lbp.3D.k_gldm_DependenceNonUniformityNormalized |  |
|  | T2_peri_wavelet.LHH_firstorder_Median |  |
|  | T1C_1_original_glcm_ClusterShade |  |
| Tumor region  +habitats  +peritumor | T2_reg_square_firstorder_Minimum |  |
|  | T1C_reg_logarithm_firstorder_Kurtosis |  |
|  | T2_reg_wavelet.LLL_firstorder_Skewness |  |
|  | T1C_3_original_glcm_Correlation |  |
|  | T1C_2_original_glrlm_LongRunLowGrayLevelEmphasis |  |
|  | T2_reg_squareroot_glcm_ClusterShade |  |
|  | T2_1_original_firstorder_Maximum |  |
|  | T1C_1_original_glcm_Autocorrelation |  |
|  | T2_3_original_ngtdm_Busyness |  |
|  | T1C_peri_lbp.3D.m2_glszm_GrayLevelVariance |  |
|  | T1C_reg_lbp.3D.m2_firstorder_Skewness |  |
|  | T2_reg_squareroot_glszm_LargeAreaHighGrayLevelEmphasis |  |
|  | T2_reg_lbp.3D.k_glszm_GrayLevelVariance |  |
|  | T2_peri_wavelet.LLH_glcm_ClusterShade |  |
|  | T2_reg_log.sigma.5.0.mm.3D_firstorder_Skewness |  |
|  | T1C_reg_log.sigma.4.0.mm.3D_firstorder_Skewness |  |
|  | T1C_peri_lbp.3D.k_glszm_SizeZoneNonUniformityNormalized |  |
|  | T1C_reg_wavelet.HLH_glrlm_LongRunHighGrayLevelEmphasis |  |
|  | T2_peri_wavelet.LHH_firstorder_Median |  |
|  | T1C_1_original_glcm_ClusterShade |  |
|  | T1C_peri_lbp.3D.k_gldm_DependenceNonUniformityNormalized |  |
